# The Treatment of Some Common Affections of the Feet

**Published:** 1906-10-27

**Authors:** Malcolm Morris

**Affiliations:** Consulting-Surgeon to the Skin Department, St. Mary's Hospital, Lecturer on Skin Diseases, London School of Clinical Medicine


### Recent Clinical Methods.

### THE TREATMENT OF SOME COMMON AFFECTIONS OF THE FEET.

By Malcolm Morris, F.R.C.S.(Edin.), etc., Consulting-Surgeon to the Skin Department, St. Mary's Hospital, Lecturer on Skin Diseases, London School of Clinical Medicine.

*Specially reported for* The Hospital.

I purpose to-day to say a few words about the common affections of the feet, which interfere with people's comfort, and prevent them from walking about and from attending to their duties in life. I propose to exclude those diseases of the feet which might be called general constitutional diseases which affect the feet, such as gout. For every one will admit that an attack of gout affecting the big toe so that a person cannot walk about with any degree of comfort could not be called a disease of the foot, and so with other diseases of the same kind. I also propose to exclude certain diseases which affect other parts of the body and only incidentally happen to affect the feet. I am concerned now with those conditions which we all see and know, but which are peculiar to the feet. I start with the condition called tender feet.

### Tender Feet.

There are a great many individuals who, when they get up in the morning, have the skin of the feet absolutely normal until they walk a certain distance, and then the feet become hot, extremely irritable, tender, sometimes worse than that. The feet swell, the skin becomes red and irritable, and when the person has an opportunity of taking off his boots and socks he sits, with a glee that is almost difficult to imagine, scratching the feet. It is a condition very well known indeed to volunteer medical officers, who have to look after men engaged in sedentary occupations all the year, but go volunteering in the summer. They suddenly find themselves walking much more than they are in the habit of doing, and the feet become swollen, red, irritable, blistered and sore—the "Volunteer" condition of feet. Medical officers of the army, especially those who practise in the tropics, know what extreme difficulties they have with individuals who suffer from tender feet. This painful tender condition is frequently accompanied, or may be caused by excessive sweating, thus bringing to bear an additional factor. How are we to help the volunteer and soldier with his hot, burning, painful feet, and the civilian who may be told by his medical adviser that a walk from the suburbs to the town is good for his health, but who when he walks gets his feet so swollen and painful that he is unable to attend to his duties during the day? The remedy for this particular trouble is the application of certain powders to the feet, which will relieve these symptoms in many cases and give the greatest possible comfort.

We divide the cases into two classes: those in which we have simply heat, redness and irritability, perhaps drying and peeling of the skin, and those in which the predominant symptom is sweating. It does not matter in the least whether the sweating is objectionable, with a bad odour or not. If there is excessive sweating the skin of the feet will become soft and sodden, and easily inflamed by walking. The powders may be divided into three groups:

I. Those which are soluble in water, and therefore equally soluble in the sweat of the feet. The powder which is soluble in water is boracic acid. It is sold in an exceedingly good preparation known as "Sanitary Rose Powder," made by Woolley, a chemist in Manchester. This is simply very finely triturated boracic acid, it is soluble in water and sweat, and is useful in cases in which there is excessive sweating, with or without a bad odour.

This is an exceedingly common condition with young servant girls and shop-girls, who very often lose their situations on account of the odour. Boracic powder dusted into their stockings will modify this.

II. Powders which are insoluble in fluid or in sweat, of which starch powder or oxide of zinc powder is a type.

III. Mixed powders—partly soluble and partly insoluble. On the whole for Volunteers and Army men and others who have much walking to do the mixed powder is the best of the three. We make the prescription in this way:—

| | |
|---|---|
| Salicylic acid (very finely powdered) ... | 1 drachm |
| Boracic acid (finely powdered) ... ... | 1 ounce |
| French chalk (carefully prepared) to ... | 4 ounces |

This is a powder which to a large extent is used in European armies for the prevention of tender sore feet.

Another powder which is worth noting is:—

| | |
|---|---|
| Salicylic acid ... ... ... ... ... | 1 drachm |
| Oleate of zinc (powdered) ... ... ... | 1 ounce |
| Talc or French chalk to ... ... ... | 4 ounces |

This makes a soapy-like powder exceedingly pleasant to the skin. Part of the boracic acid in the first prescription would be soluble in sweat. The chalk and oleate of zinc are not soluble, and would render the skin soft and comfortable, and remove the heat and redness.

Volunteers, who intend to come into camp from a sedentary occupation, should be informed that it is necessary to prepare their feet for three or four weeks beforehand, by washing them morning and night daily in tepid water with an antiseptic soap. The best soap is ichthyol superfatted soap. If that cannot be procured, carbolic or other antiseptic soap should be employed. The feet should be dried carefully and bathed afterwards in a lotion consisting of one part of spirits of wine and one part of hamamelis. The feet should be bathed with this to harden the skin, then dried and the powder placed inside the socks.

The method of rubbing in ointment to overcome this condition of sensitive feet is not so useful as that just mentioned.

### Habitually Cold Feet and Chilblains.

Many persons in various conditions of life, even on a warm summer day, complain that their feet are cold, and as soon as the weather begins to get colder and winter comes on these persons get chilblains. This is not a condition to be considered trifling, but one which should have the best medical advice. A young person who suffers habitually each winter with chilblains is potentially in a condition of danger for the rest of life. If the chilblains are neglected and the condition allowed to go on untreated the persons affected become more liable to the infection of tuberculosis and other infections. Their vitality is lowered, and they suffer each winter from the want of exercise on account of the pain from the discomfort of chilblains, and grow up to be feeble and liable to infection. I have asked many individuals who have suffered from chilblains in early life what their general health has been like since, and I have found almost invariably the same answer—delicate and out of sorts. This need not happen if the malady is properly treated during their early years. Chilblains is not simply a local disease indicating lowered vitality, although after the most careful examination nothing abnormal may be found in the heart or elsewhere, except this feeble circulation in the extremities. Sir B. W. Richardson has written a good deal on the character of the individual who suffers from chilblains. I have never been able to discover that it is a disease belonging to any particular type of individual. It seems to me largely an hereditary tendency.

Is there any particular definite line of treatment to adopt in these cases? The treatment depends on the severity of the condition. If the chilblains are slight, slight remedies are all that are necessary to restore the circulation to equilibrium. On the other hand, if they are severe and ulcerous and the child is entirely crippled, stronger and more energetic measures must be used. In the slight cases a careful investigation as to the digestive conditions —whether the food is taken properly or whether it consists of the right materials—should be made. Many of the children in semi-public schools of an elementary character—charity schools and such-like —suffer terribly from this complaint. I have been asked to inspect such schools and to report as to why such a large proportion of children were suffering from chilblains. In one particular institution I found the infirmary full of lads unable to walk on account of ulcerating chilblains. Nothing could be found wrong with the vital organs or in the quantity of food supplied, but there was a deficiency in the right sort of food. There was not a sufficient quantity of sugar, and the mere addition to the dietary of treacle or other sugary food was enough to make an enormous difference in the following winter. It is rather the fashion to think that whenever anything is wrong with the skin sugar should be discontinued, and to blame the outbreaks to a too lavish attention to the tuck-shop. I think it is essential that these children should have a sufficient quantity of sugar to keep up the normal heat, and that in this condition sugar should be increased rather than diminished. If anything has to be cut off, probably the meat portion of the dietary might be slightly reduced, because these chilblainy children have sluggish digestion and very often do not digest meat properly.

In ulcerative cases, besides medicines and the improved dietary, certain new methods of treatment are particularly useful. The radiant heat-bath will very often prevent chilblains. This applied for a limited number of days before the winter comes on will very often be sufficient to raise the circulation sufficiently to prevent the child from getting chilblains. Another method worth mentioning is the high frequency or constant current, which is particularly useful in young people who have a tendency to this condition. As regards drugs, it is very difficult to say whether any drugs have a specific action on the peripheral circulation. In some individuals some drugs are successful and in others they fail. One drug which has been mentioned by Sir A. Wright is unquestionably one which every practitioner should try in these cases of feeble circulation and chilblains—chloride of calcium. Wright has pointed out that the coagulability of the blood in these individuals is extremely slow, and chloride of calcium tends to improve this. I have tried this on many occasions and have found that it is not wise to continue the drug over too long a period. In a child of ten or twelve years of age with chilblains I would give 10 grains of chloride of calcium three times a day, but only for five or six days at a time, and in a very fair proportion of cases it is extremely useful. I do not say that it is a specific because in some it seems to fail entirely.

Another drug which has a good effect on the vasomotor nerves is one I have used for many years, and which in a large proportion of cases does good service—ichthyol. Internally, it is found beneficial to begin it in the autumn and continue it through the winter. In a child of ten I should begin with 2½ grains three times a day, giving it immediately after the meals. It can be given in the form of pill or tablet, or capsule—ichthyol is extremely disagreeable to take. Those children who cannot swallow pills or tablets must be instructed how to do so. The dose varies in different individuals; if the condition persists into middle and late life the dose must be increased to get the physiological effect. You can give up to 20 or even 30 grains three times a day. You will be enabled to tell the physiological dose that the individual can tolerate by noticing when the drug repeats. It is necessary then to curtail it.

Cod liver oil is often used in these cases. My experience is that it does not usually agree with children who suffer from chilblains. It repeats and makes them sick. I do not believe there is any peculiar advantage in cod liver oil over olive oil. Cream does good in these cases, but if you suggest this as a remedy to parents they think it is unnecessarily extravagant. As a matter of fact, it is concentrated food in a palatable form, in which these "chilblainy" individuals can easily digest. Several excellent maltine preparations are of great advantage. It is the fashion among some medical men to administer iron and arsenic or quinine either alone or combined in these cases. My experience is against these in any form. The hypophosphites are extremely useful.

Local Treatment of Chilblains.

In the erythematous stage, when chilblains are burning and extremely irritable very often it is only necessary to treat them as we do the other burning hot feet by washing with soap and afterwards stimulating the circulation with tepid water and spirit lotions, and then placing powder inside the socks. If the chilblain is actually formed more stimulating lotions are required—one of the best being the simple linimentum saponis. In the early stage applications of this painted on the foot and allowed to dry may be successful, but if there is much irritation, use with it equal parts of belladonna linament. If that does not quickly relieve try a solution of ichthyol externally, made by taking 2 drachms of ichthyol and mixing it with 2 oz. of rosewater. When this is painted on the chilblain it relieves the itching and burning. Formerly these poor sufferers were subjected to horrible tortures such as beating the chilblains with a holly bush until they bled—horrible relics of a barbarous age. For ulcerated chilblains one of the best remedies is the compound tincture of benzoin. In dealing with the question of local treatment one has to consider whether the individual is kept at rest or is still able to get about. It is obvious one can use more effective remedies if the patient is at rest. If patients are laid up, ointments are useful for ulcerated chilblains, for blisters from swelling feet, and for the form of eczema which occurs between and around the toes. The following zinc cream is a useful application. It consists of:—

| | |
|---|---|
| Oxide of zinc ... ... ... ... | 7 drachms |
| Lanoline ... ... ... ... ... | 1 drachm |
| Olive oil ... ... ... ... ... | 1 ounce |
| Lime water ... ... ... ... ... | 1 ounce |

The way to make it is to mix the olive oil and the lanoline with a little heat and add the lime-water to make an emulsion, then very slowly to add the oxide of zinc. This makes a soft creamy substance. It should be applied pretty freely spread on lint or linen with a layer of cotton wool outside and kept on with a light bandage. The dressing should be done night and morning. To this cream as a basis many drugs can be added. If there is much irritation add camphor ½ drachm. We may add ichthyol ½ a drachm. The ichthyol liquefies the cream too much and separates the lime-water; therefore, it is necessary to take out the lanoline if we are going to add the ichthyol. Another combination is Friar's Balsam 1 drachm to 3 ounces of the paste. If that application fails, which, of course, like every other remedy fails at times, the preparations of tar may be used. It is difficult to say which preparation of tar is the best. In the non-ulcerating conditions it is better to use a lotion of liquor picis carbonis in the proportion of 2 drachms to 8 oz. of water to be used as a wash. If there is ulceration use tar in the form of the ointment. The oleum Rusii is a good preparation of tar. It must be used very weak, otherwise it causes considerable pain. One drachm of this may be added to 3 ounces of the cream, or may be applied with equal parts of vaseline and starch, one ounce of each. The common corn, hard or inflamed callosities, or thickening of the soles of the feet, may be associated with chilblains, the result being great pain in walking, which is greatly improved by some preparations of sulphur. Sulphur is useful also in the common eczematous and blistered condition produced either by heat or cold. Provided that there are no ulcerations very finely powdered sulphur may be dusted in the sock. For the inflamed corn ordinary simple sulphur ointment applied night and morning while the patient is resting will afford relief. The sulphur ointment is inappropriate if the patient is going about. Lead in its various forms is particularly useful, especially so for the non-ulcerating varieties. The ointment of the subacetate of lead and glycerine is very useful for the ulcerating forms. At first it should be diluted 1 part of the ointment to 2 parts of lanoline, and afterwards equal parts, and at last the pure ointment.

In-growing Toenail.

The treatment of this exceedingly painful and troublesome malady is deserving of a few words. Surgeons seem to differ much about in-growing toenail. Many recommend its removal at once. That, I think, ought to be the last resource. The unsatisfactory part of the removal is that the nail grows rough and thickened and is apt to be extremely sensitive and painful, although there is no actual in-growing. People suffer from this trouble because they do not cut their nails properly. Cutting the nails round allows the nail to get underneath the skin. The nail grows into the surrounding skin and becomes painful on the slightest pressure. The toenails should be cut square. When the pain is felt a small piece of tin foil placed below the nail would prevent the nail growing into the skin. If granulations are small applications must be made on the principles which I have mentioned in regard to hot and tender feet. If the granulations are large let the patient rest for a time and touch the granulations with nitrate of silver, copper, or other caustic. The nail should not be removed until all other measures have entirely failed.

Corns.

Everybody knows that what is sold in shops as a corn cure is salicylic acid. If strong salicylic acid is to be used the individual should lie up for a few days, keep quiet and use no friction of the part until the corn is actually destroyed. The best preparation consists of ½ drachm of salicylic acid to 4 drachms of collodion, to which is added a little cannabis indica. The corn should be pared down as far as possible with a sharp knife, taking every possible precaution not to cause bleeding. Another useful application is a 10 per cent. pyrogallic ointment. This as a rule will remove the corn quickly. The interesting thing about it is that it has a selective action and picks out the diseased epidermis. When the skin becomes normal after this treatment advise the patient to pay the greatest attention to the details of the socks. Shoes need not necessarily be made such hideous things as are advertised as "nature-formed shoes," which indeed are not like nature at all. All that is required is free action of the toes and sufficient width at that part, but need not be wide at the extremity of the foot. I do not think broad-tipped shoes have any advantage in the prevention of corns. A great deal depends on accurately fitting socks. Treat the tender skin on the same general principles with hardening lotions and powders so as to prevent their return.

Many people suffer from soft corns between the toes. The first thing in treatment is to try to convert the soft corn into a hard one. Treat it with powders and hardening lotions until the part becomes perfectly dry, and then it can be got rid of by means of salicylic acid or by the application of pyrogallic acid or acetic acid. Acetic acid by itself will not get rid of the corn. Apply it to the little hard mass, and then before the acid is dry apply nitrate of silver. The acetic acid will carry this right through the whole of the tissue much deeper into the epidermal tissue than any solution, and in a few days the hard mass will peel.

